# Defending subjective inflation: an inference to the best explanation

**DOI:** 10.1093/nc/niaa025

**Published:** 2020-12-12

**Authors:** J D Knotts, Matthias Michel, Brian Odegaard

**Affiliations:** n1 Department of Psychology, University of California, Los Angeles, 502 Portola Plaza Los Angeles, CA 90095, USA; n2 Centre for Philosophy of Natural and Social Science, London School of Economics and Political Science, Houghton Street London WC2A 2AE, UK; n3 Consciousness, Cognition & Computation Group, Centre for Research in Cognition & Neurosciences, Université Libre de Bruxelles (ULB), 50 avenue F.D. Roosevelt CP191 B–1050, Bruxelles, Belgium; n4 Department of Psychology, University of Florida, 945 Center Dr. P.O. Box 112250 Gainesville, FL 32603, USA

**Keywords:** consciousness, richness of visual experience, subjective inflation, perceptual confidence, filling-in, signal detection theory

## Abstract

In a recent opinion piece, Abid (2019) criticizes the hypothesis that subjective inflation may partly account for apparent phenomenological richness across the visual field and outside the focus of attention. In response, we address three main issues. First, we maintain that inflation should be interpreted as an intraperceptual—and not post-perceptual—phenomenon. Second, we describe how inflation may differ from filling-in. Finally, we contend that, in general, there is sufficient evidence to tip the scales toward intraperceptual interpretations of visibility and confidence judgments.

## The Central Issues for Inflation, and Re-Appealing to Inference to the Best Explanation

We previously argued that the debate about whether visual phenomenology is rich or sparse (Lamme 2003, 2004; Kouider *et al.* 2010; Block 2011; Phillips 2018; Ward 2018) may benefit from considering a psychological phenomenon referred to as subjective inflation (Knotts *et al.* 2018; Odegaard *et al.* 2018). Briefly, subjective inflation describes the finding that human observers exhibit liberal detection biases and overconfidence in their peripheral and/or unattended visual field, despite matched objective task performance for central and/or attended stimuli (Rahnev *et al.* 2011; Solovey *et al.* 2015; Li *et al.* 2018; Odegaard *et al.* 2018). We suggested that these findings may help explain the apparent richness we experience across the entire visual field, despite known physiological and behavioral deficits in the unattended periphery.

In a recent article, Abid (2019) points out that it is ambiguous whether the psychophysical findings supporting inflation are genuinely perceptual (or intraperceptual) as opposed to cognitive (or post-perceptual) in nature (Phillips 2011; Witt *et al.* 2015). While the inflation interpretation assumes that these effects are intraperceptual, Abid provides alternative post-perceptual interpretations, which he implies should be at least as compelling as their intraperceptual counterparts. Given these ambiguities, he suggests that we ought to remain agnostic about whether inflation effects have anything to do with phenomenology.

Abid also suggests that inflation posits an unfounded connection between decisional processes and phenomenology. According to him, this problem is orthogonal (3) to the problem of determining whether inflation is an intraperceptual or post-perceptual phenomenon. However, shortly after calling the two issues orthogonal, he shows that the former ultimately boils down to the latter:

If … there is some additional argument or evidence to be given in favor of the claim that there is a decisional component to perception, then it ought to be spelled out explicitly … Although the notion of a perceptual decision does play a central role in signal detection theory, the notion is ambiguous between a detection or discrimination process that is intraperceptual and one that is post-perceptual. (4)

Accordingly, despite his emphasis that the decisional issue is precisely what is under dispute (5; see also his article’s title), his arguments focus primarily on the issue of intra- vs. post-perceptual ambiguity. Therefore, we focus on this issue here, which, we agree with Abid, is critical for inflation.

Since neither the intra- nor post-perceptual interpretation of a given behavioral effect can be directly decided by empirical evidence at the moment (Block 2007; Phillips 2011), we approach this debate about inflation effects as we have previously (Knotts *et al.* 2018), in the context of inference to the best explanation (Harman 1965; Block 2007). Specifically, we consider (i) whether there is sufficient evidence to tip the scales toward either the intra- or post-perceptual interpretation of relevant signal detection theory (SDT) criterion shifts, (ii) whether, assuming that these criterion effects are intraperceptual, inflation is different from filling-in, and (iii) whether there is sufficient evidence to tip the scales toward either the intra- or post-perceptual interpretation of visibility or confidence judgments.

## Are Inflation Effects Measured by *c* Intra- or Post-Perceptual?

Evidence supporting inflation includes the finding that observers tend to make higher numbers of false alarms in unattended or peripheral conditions in detection tasks that employ performance matching (Rahnev *et al.* 2011; Solovey *et al.* 2015). In SDT, this increase in false alarms manifests in the estimate of the perceptual criterion (*c*) used to distinguish signal from noise (Green and Swets 1966; Macmillan and Douglas Creelman 2004). While changes in *c* are associated with many perceptual illusions, such as the Müller-Lyer (Commons *et al.* 1991; Witt *et al.* 2015), sound-induced flash (Shams *et al.* 2000; Rosenthal *et al.* 2009), and stream/bounce (Grove *et al.* 2012) illusions, Witt *et al.* (2015) have argued that such changes cannot distinguish intraperceptual from post-perceptual biases. This distinction is critical for the inflation hypothesis in that intraperceptual biases are assumed to reflect changes in phenomenology, whereas post-perceptual biases are not.

One piece of evidence previously cited in favor of the intraperceptual interpretation of criterion effects is resistance to trial-by-trial feedback (Rosenthal *et al.* 2009; Rahnev *et al.* 2011; Solovey *et al.* 2015). The general intuition here is that if a bias is truly intraperceptual, there is no amount of explicit information to the contrary that can cause one to unsee it. In this sense, trial-by-trial feedback resistance is a sign of cognitive impenetrability, which is itself a criterion for determining whether a phenomenon is perceptual or post-perceptual (Pylyshyn 1999; Firestone and Scholl 2016). The Müller-Lyer illusion, which affects the criterion measure *c*, provides a good example (Witt *et al.* 2015). Even if one understands that a line with outward tails tends to appear longer than a nearby line of equal length with inward tails and modifies their responses accordingly, this knowledge and behavior do not eliminate the experience of the illusion (Brosvic and Finizio 1995).

In response, Abid argues that trial-by-trial feedback resistance is not necessarily evidence for the intraperceptual interpretation because post-perceptual biases could also resist trial-by-trial feedback. This point is well taken. Perhaps we can benefit from inference to the best explanation. What might a post-perceptual feedback-resistant bias look like? And, does it seem more or less plausible than the intraperceptual alternative?

Abid provides examples; he cites racist, religious, and superstitious biases (3) as being putatively both post-perceptual and feedback resistant. However, do we expect such high-level, social, and spiritual biases to show up in the types of controlled, low-level psychophysics experiments supporting the inflation hypothesis? We believe it is reasonable to assume that the answer to this question is no. One of the virtues of psychophysics is that we do not have to worry about such personal-level biases. If there are better, lower-level examples of post-perceptual feedback-resistant biases, Abid does not provide them. Thus, on this example alone, we argue that inference to the best explanation should point at least marginally toward the intraperceptual interpretation.

Debates on how to separate intra- and post-perceptual effects are not new. For instance, multisensory research has engaged similar issues: when perceptual reports for one sensory modality are modified by sensory input in another, is it due to post-perceptual bias, or a true change in phenomenology (Choe *et al.* 1975)? The field has largely settled on the intraperceptual interpretation by (i) trusting that subjective reports index phenomenology (Bertelson and Radeau 1976), (ii) demonstrating feedback resistance (Rosenthal *et al.* 2009), and (iii) anatomical evidence supporting multimodal interactions in early levels of the sensory hierarchy (Kayser *et al.* 2005, 2009; Ghazanfar and Schroeder 2006; Murray *et al.* 2016). We have made the case for the first two criteria in inflation.

Regarding the third criterion, while much work on the neural basis of inflation remains to be done, several sources of evidence indicate that criterion effects—such as those observed in inflation—can be intraperceptual. For instance, Jin and Glickfeld (2019) have used optogenetic manipulation of the excitability of V1 to change early sensory encoding in a detection task and induce liberal (or conservative) criteria without changing sensitivity (*d*′). As another example, high-prestimulus neural excitability—indexed by α and β power—can induce a liberal bias in detection tasks, as well as higher visibility and confidence ratings, while leaving sensitivity in discrimination tasks unchanged (Benwell *et al.* 2017; Samaha *et al.* 2017, 2020b). A study by Iemi and Busch (2018), which leverages opposing interpretations of criterion bias in two-interval forced choice detection and discrimination tasks, respectively, suggests that these prestimulus excitability effects on the criterion are intraperceptual. And further studies have indicated that these effects likely operate by modulating sensory evidence accumulation in the visual cortex (Kloosterman *et al.* 2019; Samaha *et al.* 2020a,b).

Finally, expectation-based detection and discrimination biases—such as those we have argued for with respect to inflation (Knotts *et al.* 2018)—are associated with processing in early visual cortex (Kok *et al.* 2013; Mostert *et al.* 2015; Pajani *et al.* 2015) and in areas as low level as the superior colliculus (Crapse *et al.* 2018). While the neural locus of inflation need not necessarily be constrained to early sensory regions, these findings provide additional evidence that the biases underlying inflation could be intraperceptual in nature.

## If the Relevant Changes in *c* Are Intraperceptual, Is Inflation Distinct from Filling-In?

Another criticism from Abid is the following: if the relevant changes in *c* are intraperceptual, then inflation is not distinct from the phenomenon of perceptual filling-in (Komatsu 2006). However, if these changes in *c* are post-perceptual, then inflation is irrelevant for phenomenology. Regardless of which option is correct, inflation fails to contribute anything meaningful to the debate.

Reiterating the operational definition of inflation may help clarify what it contributes to the debate about phenomenology. Inflation occurs when there is a difference in subjective ratings between two conditions for which objective performance is matched. On this definition alone, inflation is, at least in theory, distinct from filling-in; one can imagine there being filled-in perceptual content for which subjective judgments are not inflated. And one can imagine there being inflated subjective judgments that are not based on filled-in perceptual content. However, giving Abid the benefit of the doubt, we might hypothesize that these two phenomena always co-occur, rendering them, in practice, indistinct. If this is true, then wherever filling-in is observed, inflated subjective judgments should be observed as well.

Some preliminary evidence for filled-in representations being subjectively inflated comes from an elegant study by Ehinger *et al.* (2017), in which participants were more likely to choose a filled-in striped pattern than a veridical one when forced to indicate which of the two patterns was continuously striped. Similar evidence has been found for filled-in foveal content under low light conditions (Gloriani and Schütz 2019), but it remains an open question whether inflated subjective judgments are an intrinsic feature of filling-in, or, rather, if inflation is an independent, dissociable process. The same question can be asked of the potential relationship between subjective inflation and other peripheral mechanisms that involve summary statistics (Cohen *et al.* 2016). For example, are subjective ratings for crowded stimuli inherently inflated, as has been observed in at least one case (Odegaard *et al.* 2018)?

These questions are empirically tractable and should be investigated in future studies. But, regardless of whether inflation is ultimately an inherent feature of other known peripheral summary mechanisms or an independent, dissociable process, what is important is that it provides explanatory power for a fundamental question about the apparent richness of phenomenology: why, if peripheral vision is coarse and summarized, does it not feel coarse and summarized? It feels so because subjective evaluation of these coarse and summarized representations is systematically biased, or inflated, relative to what we would predict based on the relationship between objective and subjective judgments in typical, foveal vision. This empirically tractable operationalization is the novel evidence aimed at explaining the apparent richness of visual phenomenology (Abid 2019, 4) that the inflation hypothesis offers.

**Figure 1.**
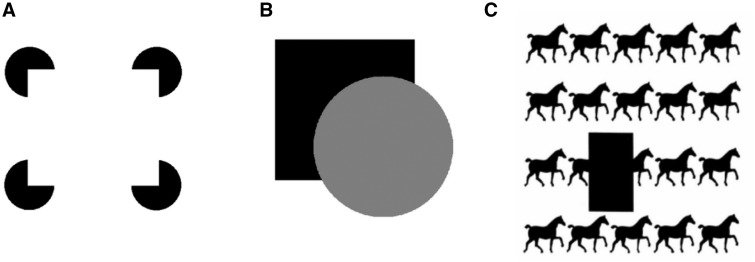
(**A**) Kanizsa square: an example of modal completion. (**B**) The partly occluded square is amodally completed and thus seems perceptually present behind the occluding disk. (**C**) Amodal completion results in the feeling of presence of a long horse behind the rectangle occluder, instead of two horses. As noted by Kanizsa (1985), these and similar examples suggest that amodal completion is partly independent from logical reasoning and expectations, thereby indicating that it is not a purely post-perceptual phenomenon (Nanay 2010).

While operational definitions are useful for understanding inflation from a behavioral standpoint, we agree with Abid that more precisely characterizing what it is like to have inflated phenomenology, and how it may differ from what it is like to experience filled-in content, is essential. The phenomenological difference between modal and amodal completions may provide a helpful analogy. Modal completion involves the subjective visual perception of filled-in contours and surfaces. For example, one can clearly see filled-in, illusory contours between the Pac-Men-like shapes at each corner of a Kanizsa square (Fig. 1A). In modal completion, completion operates by a form of filling-in that has modal features (e.g. visual features). In amodal completion, on the other hand, the completed parts are not subjectively visualized—which is why it is amodal—but are still visually represented, giving rise to a visual sense of presence of occluded figures (Fig. 1B;  Michotte and Burke 1951; van Lier and Gerbino 2015; Thielen *et al.* 2019).

Importantly, evidence suggests that amodal completion is at least partly a perceptual process: it can be cognitively impenetrable (Kanizsa 1985; Ekroll *et al.* 2016, 2017; Fig. 1C); it occurs relatively early on in visual processing (Sekuler and Palmer 1992; Murray *et al.* 2001; Chen *et al.* 2009); occluded objects pop out as do their unoccluded counterparts in visual search (Rensink and Enns 1998); and amodally completed objects can induce adaptation effects that are similar to those induced by their unoccluded counterparts (Weisstein *et al.* 1972; Fang and He 2005).

Although amodal completion and subjective inflation probably depend on different neurocognitive mechanisms, we suggest that, at the phenomenological level, perceptual inflation may feel somewhat like amodal completion. Both phenomena involve a perceptual sense of presence that is distinct from active filling-in, or conscious perception of qualitatively visual features, such as colors. In that sense, one can phenomenologically characterize subjective inflation in the visual periphery as a feeling of presence of visual features, similar to the feeling of presence elicited by looking at the square in Fig. 1B, or the sausage horse in Fig. 1C. In much the same way that amodal completion is thought to be a perceptual phenomenon and is different from modal completion, we hold that subjective inflation is a perceptual phenomenon, distinct from filling-in.

Of course, this does not mean that filling-in does not contribute to the overall sense of phenomenal richness. Presumably, the two processes would be complementary (e.g. as we have described for the studies by Gloriani and Schütz 2019; Ehinger *et al.* 2017). Furthermore, the distinction between modal and amodal completion does not, in and of itself, provide support for the idea that filling-in and inflation are, in practice, dissociable processes. As mentioned above, it may be that filled-in representations always display the operational signature of inflation. The analogy to modal and amodal completion is simply intended to provide a more intuitive sense of how the two processes could differ in terms of their respective contributions to phenomenology.

## Are Subjective Judgments Intra- or Post-Perceptual?

Abid’s last major criticism of inflation regards the validity of subjective judgments as measures of phenomenology. His primary challenge is that sources of information other than introspection about visual phenomenology may contaminate subjective judgments. This is analogous to his argument about post-perceptual interpretations of SDT criterion shifts. For example, regarding confidence judgments, he states that A student deep in the thick of Cartesian skepticism may lack confidence in all their perceptual judgments, but it would be preposterous to claim that reading Descartes might render one visually impaired (4).

We agree and suggest that precisely what makes such a claim—which amounts to an odd type of Orwellian postdiction (Phillips 2011)—preposterous is that Abid is describing a different type of confidence judgment than what is typically asked for in perceptual tasks. One amounts to an abstract, philosophical position, while the other is a simple metacognitive judgment of task performance. If a Cartesian skeptic with a normally functioning visual system repeatedly rates zero confidence in their performance on a task that they are performing significantly above chance, then the simplest explanation is that they are failing to comply with task instructions. But we do not need to worry about such cases as long as we do our due diligence as experimenters and provide clear instructions. If, on the other hand, Abid suggests that study participants should not be trusted to follow task instructions in general, then his argument overgeneralizes to psychological research as a whole.

Abid provides other examples of a similar flavor. For instance, he suggests that the overconfidence observed in crowding (Odegaard *et al.* 2018) may be due to participants knowledge that they can often discriminate objects surrounded by other objects in non-laboratory settings: they simply need to move their eyes and fixate them. While we cannot definitively rule out this possibility, is it the best explanation of the observed phenomenon? This interpretation fails to acknowledge that overconfidence was observed for crowded stimuli relative to non-crowded stimuli. Why would the knowledge that eye movements can improve perceptual inference apply more to the crowded condition? Again, Abid offers a tenable but uncompelling example of a post-perceptual bias that could contaminate an intended measure of phenomenology. Inference to the best explanation again favors the simpler interpretation that participant’s confidence judgments meaningfully reflect what they see.

While Abid explicitly targets studies using visibility (Rahnev *et al.* 2011) and confidence ratings (Odegaard *et al.* 2018) cited as evidence in support of subjective inflation, his arguments generalize to all studies that use confidence and visibility judgments as measures of phenomenology, by groups on all sides of many key debates in consciousness science (e.g. Dehaene and Naccache 2001; Landman *et al.* 2003; Ramsøy and Overgaard 2004; King and Dehaene 2014; Bronfman *et al.* 2014; Maniscalco and Lau 2016; Webb *et al.* 2016; Ward *et al.* 2016; Matthews *et al.* 2018). Ultimately, any result in the psychology of perception relying on any kind of reports can be re-interpreted as post-perceptual, provided that one is willing to assume the existence of sufficiently complex post-perceptual biases. However, it would be incorrect to conclude, for instance, that visibility judgments never tell us anything about phenomenology simply because alternative post-perceptual explanations of these judgments are conceivable, or because those judgments can sometimes be dissociated from phenomenology. To avoid this overgeneralization, we suggest that subjective judgments should be considered meaningful indicators of phenomenology until proven guilty of post-perceptual influences.

But this is not to say that subjective measures are simply better than nothing. In addition to their face validity for assessing phenomenology, recent work explains the utility and relevance of using confidence ratings in paradigms where performance is matched to probe conscious awareness of phenomenological content (Morales *et al.* 2019), and at least some perceptual findings using confidence and visibility judgments cannot be accounted for by post-decisional cognitive/memory biases (Samaha and Denison 2020). Researchers can also analyze visibility and confidence judgments within the framework of SDT (Maniscalco and Lau 2012; Galvin *et al.* 2003), which offers rigorous tools for quantifying the effects of biases in experimental settings. This type of empirical rigor stands in contrast to recent appeals to use open-ended picture drawing and verbal descriptions to probe phenomenology (Haun *et al.* 2017). While such approaches are not necessarily without merit, they are just as susceptible to post-perceptual re-interpretation, while lacking the benefits of fitting within a quantitative framework. Thus, if the goal is to have a true science of conscious awareness that involves testable predictions and quantifiable measures, confidence and visibility judgments currently represent our best option, as evidenced by their pervasive use in consciousness research. Of course, this is not to say that they represent a perfect, linear index of phenomenology, as we have acknowledged previously (Knotts *et al.* 2018; Odegaard *et al.* 2018; Michel 2019).

## Conclusion and Future Directions

Overall, we appreciate Abid’s appeal to skepticism given the current limits on what we can unequivocally infer about consciousness from empirical investigation (Block 2007; Phillips 2011). However, we agree with Block (2007) that given this difficulty, progress in our understanding of consciousness hinges critically on inference to the best explanation. In this regard, we have argued that shifts in both the SDT criterion and subjective judgments that have been cited as evidence for subjective inflation (Knotts *et al.* 2018; Odegaard *et al.* 2018) are best explained as being intraperceptual. We have also attempted to clarify, by way of analogy to amodal completion, the putative qualitative nature of inflated phenomenology.

There are still many questions about inflation that need answering. For example, why does inflation occur? Despite Abid’s puzzling claim to the contrary (Abid 2019, 6), we have already written on this issue (Knotts *et al.* 2018; Odegaard *et al.* 2018), suggesting that it may be linked to self-consistent perception (Stocker and Simoncelli 2007; Luu and Stocker 2016), or could be due to reliance on priors (Kouider and Dupoux 2004; Kouider *et al.* 2007) and/or summary representations (Cohen *et al.* 2016) when information is sparse. However, arguments can only take this debate so far in the near future. What we need is more data to test whether or not this phenomenon extends beyond what has been shown thus far, including the degree to which it does (or does not) extend across different perceptual domains, the degree to which it is (or is not) supported by paradigms requiring different degrees of decisional complexity (detection vs. discrimination vs. identification; Odegaard and Lau 2016), the degree to which it does (or does not) operationally dissociate from filling-in, and the degree to which it may be compatible (or not) with contemporary ideas and theories about why we have phenomenology at all (Gershman 2019; Lau 2019). The full explanatory power of inflation can be explored in tests that we have outlined previously (Knotts *et al.* 2018; Odegaard *et al.* 2018), and we look forward to seeing whether this idea can continue to provide insight regarding a historically difficult scientific problem.
